# PPARα-independent effects of nitrate supplementation on skeletal muscle metabolism in hypoxia^☆^

**DOI:** 10.1016/j.bbadis.2018.07.027

**Published:** 2019-04-01

**Authors:** Katie A. O'Brien, James A. Horscroft, Jules Devaux, Ross T. Lindsay, Alice Strang Steel, Anna D. Clark, Andrew Philp, Stephen D.R. Harridge, Andrew J. Murray

**Affiliations:** a Department of Physiology, Development & Neuroscience, University of Cambridge, Cambridge, UK; b Centre for Human and Applied Physiological Sciences, King's College London, London, UK; c School of Sport, Exercise and Rehabilitation Sciences, University of Birmingham, Birmingham, UK; d Diabetes and Metabolism Division, Garvan Institute of Medical Research, Darlinghurst, Australia

**Keywords:** Muscle, Metabolism, Hypoxia, Nitric oxide, Fatty acids

## Abstract

Hypoxia is a feature of many disease states where convective oxygen delivery is impaired, and is known to suppress oxidative metabolism. Acclimation to hypoxia thus requires metabolic remodelling, however hypoxia tolerance may be aided by dietary nitrate supplementation. Nitrate improves tissue oxygenation and has been shown to modulate skeletal muscle tissue metabolism via transcriptional changes, including through the activation of peroxisome proliferator-activated receptor alpha (PPARα), a master regulator of fat metabolism. Here we investigated whether nitrate supplementation protects skeletal muscle mitochondrial function in hypoxia and whether PPARα is required for this effect. Wild-type and PPARα knockout (PPARα^−/−^) mice were supplemented with sodium nitrate via the drinking water or sodium chloride as control, and exposed to environmental hypoxia (10% O_2_) or normoxia for 4 weeks. Hypoxia suppressed mitochondrial respiratory function in mouse soleus, an effect partially alleviated through nitrate supplementation, but occurring independently of PPARα. Specifically, hypoxia resulted in 26% lower mass specific fatty acid-supported LEAK respiration and 23% lower pyruvate-supported oxidative phosphorylation capacity. Hypoxia also resulted in 24% lower citrate synthase activity in mouse soleus, possibly indicating a loss of mitochondrial content. These changes were not seen, however, in hypoxic mice when supplemented with dietary nitrate, indicating a nitrate dependent preservation of mitochondrial function. Moreover, this was observed in both wild-type and PPARα^−/−^ mice. Our results support the notion that nitrate supplementation can aid hypoxia tolerance and indicate that nitrate can exert effects independently of PPARα.

## Highlights

• Hypoxic exposure suppresses mitochondrial respiration in mouse skeletal muscle.
• Loss of citrate synthase activity suggests decreased mitochondrial content.
• Dietary inorganic nitrate partially protects against hypoxia-induced changes.
• Nitrate protects independently of peroxisome proliferator-activated receptor α.

## 1 Introduction

Tissue hypoxia is a key feature of many pathologies, including cardiovascular disease, respiratory diseases and many cancers [1], and is also experienced by healthy individuals at high altitude. The physiological response to hypoxia includes changes that improve convective oxygen delivery [2], however it is increasingly recognised that changes in oxygen utilisation at the tissue level can also occur. A fall in the capacity for oxidative metabolism, including fatty acid oxidation, occurs in the skeletal muscle of humans and rodents following prolonged exposure to hypoxia [3]. A purported key player in regulating this response is peroxisome proliferator-activated receptor α (PPARα), a transcriptional regulator of fatty acid metabolism in heart, liver and skeletal muscle [4]. PPARα is downregulated in hypoxic cells under the action of hypoxia-inducible factor 1 (HIF1) [5], whilst its expression falls in lowlander muscle following acclimatisation to high altitude [6]. Meanwhile, in hypoxic rodent heart, the transcriptional response to hypoxia includes downregulation of PPARα target genes, and a switch in substrate preference away from fatty acid oxidation [[7], [8], [9], [10]].

Such changes in oxidative metabolism may occur alongside an increased reliance on glucose metabolism, including increased glycolysis [[11], [12], [13]], potentially improving the efficiency of oxygen utilisation (ATP produced per O_2_ consumed) and thereby matching tissue oxygen demand to the diminished supply, but this may also limit the capacity for ATP synthesis. In hypoxic rats, metabolic changes were associated with lower ATP levels in skeletal muscle, in comparison with normoxic rats [14]. These changes, however, were prevented in rats that received a moderate dose of sodium nitrate via the drinking water, protecting muscle ATP levels [14].

Inorganic nitrate (NO_3_^−^) can be consumed by humans as part of a normal, healthy diet with high levels present in green leafy vegetables and beetroot [15]. At a whole body level, nitrate supplementation has been shown to decrease the O_2_ cost of exercise in healthy subjects in both normoxic and hypoxic conditions [[16], [17], [18], [19], [20]], in a clinical population suffering from hypoxia as a result of chronic obstructive pulmonary disease [21], and also in subjects with decreased O_2_ carrying capacity due to blood donation [22].

Once consumed, nitrate is believed to be reduced in a stepwise fashion, firstly to nitrite (NO_2_^−^) and then to nitric oxide (NO) [23, 24], adding to the endogenous production of NO from the NO synthase-dependent oxidation of L-arginine [25]. NO is a diatomic free radical that acts as a signalling molecule and is regarded to be the chief mediator of the responses observed with nitrate supplementation [26]. NO has been shown to exert numerous biological effects in relation both to the delivery of O_2_, through the well characterised mediation of vascular tone [27,28], and the utilisation of O_2_ through effects upon mitochondrial function. Whilst some of the changes in tissue metabolism exerted by NO may occur secondary to altered haemodynamic function and improved O_2_ delivery [29], studies in cultured myocytes have shown direct effects of nitrate supplementation on fatty acid metabolism via activation of the PPAR family of transcription factors by cyclic GMP (cGMP), a downstream mediator of NO activity. In vivo, nitrate supplementation thereby enhanced fatty acid oxidation capacity and mitochondrial content in the skeletal muscle of normoxic and hypoxic rodents, lowering intramuscular levels of long-chain fatty acid intermediates [14].

Elevated levels of NO metabolites have been seen in human populations native to high altitude regions in Tibet and the Andes [[30], [31], [32]], with selection of genetic variants in NO synthase genes [33]. In particular, Tibetans have high levels of exhaled NO [34] and circulating NO biomarkers, which are associated with enhanced forearm blood flow [32]. The haemodynamic effects of elevated NO production may therefore be protective at altitude, indeed in lowlanders circulating levels of nitrate, nitrite and cGMP increased during acclimatisation [35]. It is not clear though, whether the protective effects of nitrate on mitochondrial function and other aspects of energy metabolism occur at altitude, since PPARα expression and/or activity is downregulated in hypoxic skeletal muscle [6,10]. Moreover, in one Tibetan population, the Sherpa, selection for a variant in the gene encoding PPARα (*PPARA*) is associated with a lower capacity for skeletal muscle fatty acid oxidation compared with lowlanders, alongside improved mitochondrial efficiency, lower levels of oxidative stress and protection of muscle energetics at high altitude [6].

We therefore hypothesised that inorganic nitrate exerts protective effects on skeletal muscle mitochondrial function in the context of hypoxia, independent of PPARα activation. To investigate this we employed an 8-arm study design, exposing wild-type or PPARα knockout mice to normoxia or 10% hypoxia for four weeks, and supplementing them with either a moderate dose of sodium nitrate (0.7 mM) or sodium chloride (0.7 mM) as control. Mitochondrial respiratory function was measured in saponin-permeabilised skeletal muscle fibres, whilst levels of metabolic proteins and regulators were measured using immunoblotting.

## 2 Methods

Animal work was conducted in accordance with UK Home Office regulations under the Animals in Scientific Procedures Act, and underwent review by the University of Cambridge Animal Welfare and Ethical Review Committee. Procedures involving live animals were carried out by a licence holder in accordance with these regulations.

### 2.1 Study design

Mice were bred on a pure 129Ev/Sv background with 10 backcrosses [7]. The original breeding pairs were a kind gift of Frank Gonzalez (National Institutes of Health, Bethesda, MD). Male wild-type 129Ev/Sv (n = 42) and constitutive PPARα^−/−^ littermates (n = 42) were housed in conventional cages from birth in a temperature (23 °C) and humidity controlled environment with a 12 h/12 h light/dark cycle. Normoxic mice were housed under the same environmental conditions as those mice in the hypoxia chamber. Mice were fed a standardised quality-controlled diet (RM1(E)SQC), comprising 65.0% carbohydrate, 13.1% crude protein, 3.5% crude fat, 10 mg/kg nitrate, trace nitrite (Special Diet Services, Witham, Essex, UK) and initially provided with distilled water ad libitum.

**Fig. 1:**
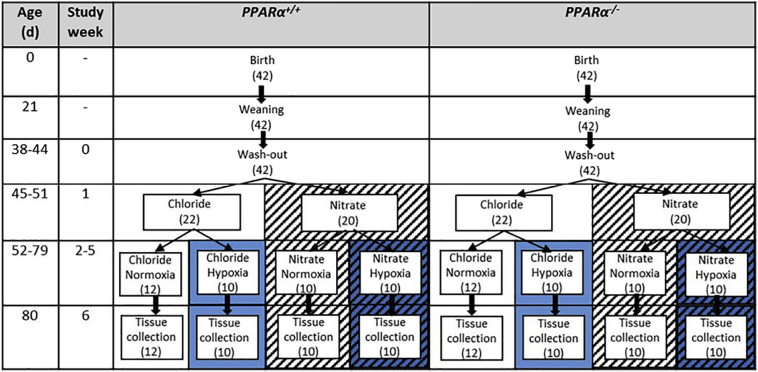
Study design. Each stage took place within the ages shown ±4 d, and the length of each stage was identical for each mouse. The left section represents wild-type (PPARα^+/+^) mice and the right section represents PPARα knockout (PPARα^−/−^) mice. The number in brackets indicates the number of mice in each group. Chloride (0.7 mM NaCl) in distilled water ad libitum; Nitrate (0.7 mM NaNO_3_) in distilled water ad libitum; Normoxia, 21% atmospheric O_2_; Hypoxia, 10% atmospheric O_2_. Background patterns reflect those used in box and whisker plots in subsequent figures.

The study design is outlined in Fig. 1. At 6 weeks of age, mice from both genotypes were randomly assigned to receive drinking water supplemented either with 0.7 mM sodium nitrate (NaNO_3_) or 0.7 mM sodium chloride (NaCl) for the remainder of the study. NaCl was used to control for any effects of sodium intake. A concentration of 0.7 mM was chosen as this has been shown to increase plasma and tissue levels of nitrogen oxides in mice [36].

One week following the initiation of sodium nitrate or sodium chloride treatment, mice were randomly assigned either to remain under normoxic conditions (21% O_2_) or to be housed in hypoxia (10% O_2_) in a chamber (PFI Systems Ltd., Milton Keynes, UK). Mice remained in these conditions for four weeks. Nitrate treatment was thus initiated 1 week prior to hypoxic exposure in order to achieve stable circulating nitrogen oxide levels. Body mass, food and water intake were measured upon initiation of treatment, and thence weekly.

Mice were killed 80 ± 4 days after birth by dislocation of the neck. From each mouse, one soleus was excised and immediately placed in ice cold biopsy preservation solution (BIOPS), comprising 2.77 mM CaK_2_EGTA, 7.23 mM K_2_EGTA, 6.56 mM MgCl_2_.6H_2_O, 20 mM taurine, 15 mM phosphocreatine, 20 mM imidazole, 0.5 mM dithiothreitol, 50 mM MES, 5.77 mM Na_2_ATP, pH 7.1 [10] for respirometry experiments (Section 2.2). Following this, the muscle fibre bundles were recovered from the Clark electrode chambers and homogenised for citrate synthase activity quantification (Section 2.3) and determination of myosin heavy chain isoform composition via SDS-PAGE (Section 2.4). The second soleus was snap-frozen in a cryovial under liquid nitrogen for western blotting to assess metabolic proteins (Section 2.5). A drop of blood taken from the tail vein was loaded into a microcuvette and haemoglobin concentration analysed using a HemoCue Hb201 Analyzer (Quest Diagnostics, Sweden).

### 2.2 Measurement of mitochondrial respiratory function

Soleus muscle fibre bundles were permeabilised using saponin as previously described [37]. Respirometry was carried out at 37 °C using a Clark-type oxygen electrode and a water-jacketed chamber with constant stirring (Strathkelvin Instruments, Motherwell, UK). Respiration rates were analysed using two substrate/inhibitor assays with each assay run in duplicate. In Assay 1, flux through the N-pathway via electron transfer system complex I was assessed initially in the LEAK state in response to the addition of 2 mM malate plus 10 mM glutamate (GM_*L*_), with OXPHOS being subsequently being stimulated through the addition of 10 mM ADP (GM_*P*_). In order to inhibit complex I, 0.5 μM of rotenone was then added, followed by the addition of 10 mM succinate to assess flux through the S-pathway via complex II (S_*P*_). In Assay 2, fatty acid oxidation capacity was assessed initially in the LEAK state through the addition of 2 mM malate plus 40 μM palmitoyl-carnitine (PalM_*L*_). Following this, oxidative phosphorylation (OXPHOS) was stimulated through the addition of 10 mM ADP (PalM_*P*_) and carbohydrate oxidation was assessed through the addition of 5 mM pyruvate (PM_*P*_). Oxygen consumption was normalised to the wet weight of tissue used (approximately 0.5–1.5 mg per assay).

### 2.3 Citrate synthase activity assay

Following respirometry, the contents of the chamber were extracted and homogenised using a Polytron (PT-10-35 GT Kinematic Inc., Switzerland) at 25,000 rpm for 30 s, with the addition of 1% Triton-×100 and 10 mg/ml protease inhibitor (Complete Protease Inhibitor Cocktail, Roche). Protein content of whole soleus muscle homogenates was assessed using Pierce BCA Protein Assay Kit (Thermo Scientific), with subsequent detection performed using a Nanodrop 2000 spectrophotometer (ThermoFisher Scientific).

Citrate synthase activity was quantified on the resulting homogenate at 37 °C as described previously [38, 39]. The assay buffer contained 20 mM Tris, 0.1 mM, 5,5′-dithiobis2-nitrobenzoicacid and 0.3 mM of acetyl CoA at pH 8.00. The reaction was initiated by the addition of 0.5 mM oxaloacetate and absorbance change at 412 nm was measured (Evolution 220, Thermo Scientific).

### 2.4 SDS-polyacrylamide gel electrophoreisis (PAGE) for soleus myosin heavy chain isoform composition

The homogenate was spun using a microcentrifuge for 10 min at 13,000 rpm. This was re-suspended in Laemmli sample buffer [40] at a concentration of 40 μl/mg protein.

Gels were cast in vertical slab mini-gel plates with a thickness of 0.75 mm. The separating gel, left to set at room temperature overnight, comprised 30% glycerol, 4% acrylamide-N-*N*′-methylene-bis-acrylamide (bis) (50:1), 0.2 M Tris (pH 8.8), 0.1 M glycine and 0.4% SDS. To prevent drying, 10% Sodium Dodecyl Sulphate (SDS) was sprayed and cling film wrapped around the top of the plates. This was removed prior to the addition of the stacking gel, which comprised 30% glycerol, 4% acrylamide-bis (50:1), 70 mM Tris (pH 6.7), 100 mM EDTA (pH 7.0), 0.4% SDS. A 0.75 mm 10-well spacer was inserted into the stacking gel immediately after pouring. This was left to set for 1 h before the spacer was removed and loading of the samples commenced. The pH of the stock solutions for both separating and stacking gels was not adjusted after these solutions were mixed. Polymerization of both gels was initiated with 0.05% of NNN'*N*′-Tetramethylethylenediamine (TEMED) and 0.1% ammonium persulfate (APS).

The gels were run utilising the Mini-PROTEAN II Cell set up (Bio-Rad Laboratories). Upper running buffer, consisting of 50 mM Tris, 75 mM glycine, 0.05% SDS and 0.1% β-mercaptoethanol, was added to the space between the two gels. Lower running buffer, containing the same constituents as the upper buffer minus β-mercaptoethanol, was added to the tank. A molecular weight marker 7 μL (Precision Plus Protein™ Kaleidoscope™, Bio-Rad) was loaded alongside 13 μL of muscle sample. The gel was then run at ~4 °C for 24 h at a constant current of 4 mA (PowerPack 300, Bio-Rad Laboratories).

Following completion of the SDS-PAGE run, the stacking gel was removed and the separating gel washed in ddH_2_O. In order to visualise and analyse the protein bands, the mini gel was stained with 20 ml Coomaisse®G250 stain, SimplyBlue™ Safestain (Invitrogen), for 1 h on a rocker. It was then washed in 100 ml ddH20 for 1 h prior to scanning using a HP Scanjet 3500c Series. The bands corresponding to myosin heavy chain isoforms, located between 150 and 250 kDa, were analysed by gel densitometry using ImageJ. From this, the proportion of type 1 and 2 fibres in each soleus sample was assessed.

Due to the inherent differences in fibre composition, extensor digitorum longus (EDL) muscle was used as a control comparison to the soleus. This was dissected from 3 month old female wild type Parkes wild type strain (PKS) mice, donated by Malcolm Logan (King's College London). Wet weight was obtained and sample buffer added with a minimum of 500 μl being required for homogenisation using a gentleMACS dissociator (Miltenyi Biotech).

### 2.5 Western blotting for soleus metabolic proteins

Whole frozen soleus was powdered and then homogenised as described previously [41]. For all antibodies excluding OXPHOS (see below), samples were boiled prior to loading. Western blotting was conducted as described previously [41]. The antibodies used were: Total OXPHOS rodent WB antibody cocktail (OXPHOS) (ab110413, Abcam), Anti-citrate synthase (CS) (SAB2701077, Sigma), mitofusin 2 (MFN2) (Cell Signalling 9482), PGC1α (AB3242, Millipore), long chain acyl coA dehydrogenase (LCAD, a gift from Prof. Jerry Vockley, University of Pittsburgh [42]), carnitine palmitoyltransferase (CPT) 1 (CPT1MIIA, Alpha Diagnostics) and pyruvate dehydrogenase kinase 4 (PDK4, a gift from Prof. Grahame Hardie, University of Dundee [43]). All primary antibodies were diluted 1:1000, with the exception of LCAD, which was diluted 1:5000. Secondary antibodies were diluted 1:10,000 and were as follows: Rabbit anti-mouse IgG (Pierce #31457), rabbit anti-sheep IgG (Pierce #31480), goat anti-rabbit (Pierce #31460). Immmobilon western chemiluminescent HRP substrate (Merck Millipore) was used to quantify protein content after IgG binding, and visualized on a G:BOX Chemi XT4 imager using GeneSys capture software (Syngene, Cambridge, UK). Density of bands corresponding to the target protein were corrected to the loading control (mouse gastrocnemius) for that blot as well as the corresponding actin band derived from Ponceau S staining.

### 2.6 Statistics

A three-way ANOVA was performed to assess the effects of hypoxia, nitrate and PPARα, as well as the interactions between these factors. The statistical approach has been described previously [44]. Initially a three-way interaction was considered for significance. If a significant three-way interaction was found, main effects and two-way interactions were disregarded and a post-hoc Tukey's HSD (honest significant difference) test performed. Pairwise comparisons of groups between which only one of the independent variables differed were considered, and the results recorded. If the three-way interaction was not significant, a post-hoc Tukey's HSD test was performed to investigate each two-way interaction. Any main effects of variables involved in the significant two-way interaction(s) were not considered, while main effects of variables not involved in significant interactions were. In instances where no three-way or two-way interactions were significant, the main effects of all three independent variables were considered. Main effects combine all four groups in each state of one independent variable and make a pairwise comparison between those combinations (e.g. all four hypoxic vs all four normoxic groups). All analyses were performed using R software (R Foundation for Statistical Computing). Differences were considered statistically significant when *P* < 0.05.

Graphs were generated using Graphpad Prism 7 software and follow a colour/pattern scheme whereby white indicates normoxia (21% O_2_), blue hypoxia (10% O_2_), block colour chloride (Cl^−^) and striped nitrate (NO_3_^−^) treated groups. In addition, the wild type (PPARα^+/+^) are separated from the knockout (PPARα^−/−^). The majority of the data (with the exception of body weights and MyHC isoform composition) is shown as minimum to maximum box and whisker plots, with the middle line representing the median and the box the interquartile range (25th to 75th percentiles), so that the full data spread can be visualized. Significant differences are indicated with black asterisks highlighting interactions between genotypes (PPARα^−/−^ vs PPARα^+/+^), blue asterisks highlighting interactions linked to O_2_ state (hypoxia vs normoxia) and orange asterisks highlighting interactions linked to treatment group (nitrate vs chloride).

## 3 Results

### 3.1 Body weights, food, water and nitrate intake and circulating haemoglobin

**Fig. 2:**
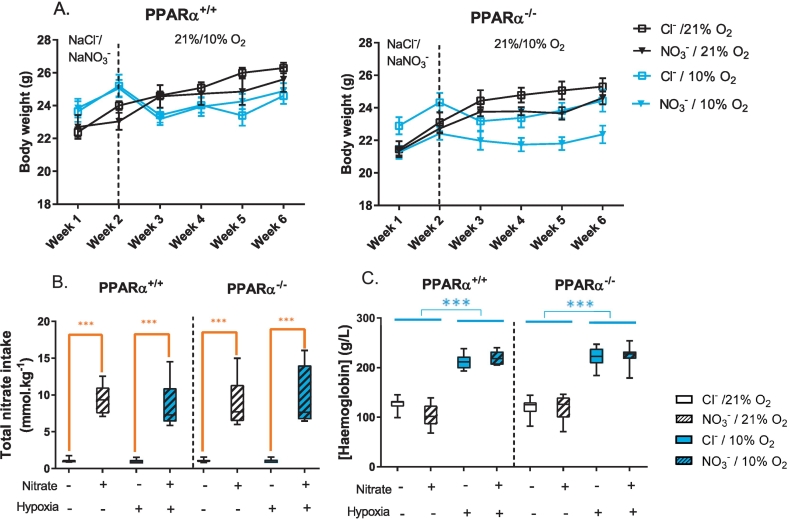
Body weights, nitrate intake and circulating haemoglobin. A) Body weights (n = 10–12 per group) on the first day of the week indicated, with dietary nitrate supplementation beginning on week 1 and hypoxic treatment beginning on week 2. Data represented as mean ± SEM. B) Cumulative nitrate (NO_3_^−^) intake from food and water throughout the study. C) Haemoglobin concentration (g/L), immediately after sacrifice. Data are represented as minimum to maximum values, n = 6–12 per group. ***P < 0.001 nitrate effect (orange) or hypoxia effect (blue).

Body weights were recorded on the first day of each week (Fig. 2A). At the initiation of supplementation with sodium chloride or sodium nitrate (Week 1), body weights did not differ between the chloride-fed and nitrate-fed groups, nor between normoxic and hypoxic groups. However, PPARα^−/−^ mice were 6% lighter than wild-type mice (*P* < 0.001, PPARα main effect). After one week, and just prior to initiation of normoxic or hypoxic exposure (Week 2), body mass did not differ between the mice that would be exposed to normoxia or those that would be exposed to hypoxia. At this time-point, nitrate-treated mice were 4% lighter than chloride-treated mice (*P* < 0.05, nitrate main effect). After one week of exposure to normoxia or hypoxia (Week 3), hypoxic mice were 5% lighter than normoxic mice (*P* < 0.001, hypoxia main effect), but neither PPARα nor nitrate treatment had any effect on body weight at this time-point. At the end of the study (Week 6), hypoxic mice remained 4% lighter than normoxic mice (*P* < 0.001, hypoxia main effect). In addition, PPARα^−/−^ mice were 5% lighter than wild-type mice (*P* < 0.01, PPARα main effect), and nitrate-treated mice were 3% lighter than chloride-treated mice (*P* < 0.05, nitrate main effect).

Neither treatment with nitrate, nor PPARα^−/−^ had any effect on food intake at any time-point, nor did they interact with any other factor (Supplementary Fig. 1). In Week 1, food intake was similar between all groups. In the first week of hypoxic exposure (Week 2), however, food intake of hypoxic mice was 34% lower than in normoxic mice (*P* < 0.001, hypoxia main effect). This difference in food intake persisted in week 3 (10% lower intake, *P* < 0.001, hypoxia main effect) and week 4 (8% lower intake, *P* < 0.001, hypoxia main effect), but food intake was the same in all groups by week 5. Water intake was similar between all groups in Weeks 1, 3, 4 and 5. In Week 2, however (the first week of hypoxic exposure) water intake was 30% lower in hypoxic mice compared with normoxic mice (*P* < 0.001, hypoxia main effect) and 10% lower in PPARα^−/−^ mice compared with wild-types (*P* < 0.05, PPARα main effect).

Over the course of the study, nitrate intake from food and water was 8.8-fold higher in nitrate-treated mice compared with chloride-treated mice (*P* < 0.001, nitrate main effect, Fig. 2B). In Week 2, total nitrate intake was decreased by 6% by hypoxic exposure in the nitrate-fed hypoxic mice (*P* < 0.001, Tukey's test of nitrate/hypoxic interaction) owing to the acute effect of hypoxic exposure on water intake, however water intake and thus nitrate intake was restored in these mice by Week 3.

Blood haemoglobin concentration [Hb], measured at the end of the study, was 85% higher in hypoxic mice than in normoxic mice (*P* < 0.001 hypoxia main effect, Fig. 2C).

### 3.2 Myosin heavy chain isoform composition and mitochondrial morphology

**Fig. 3:**
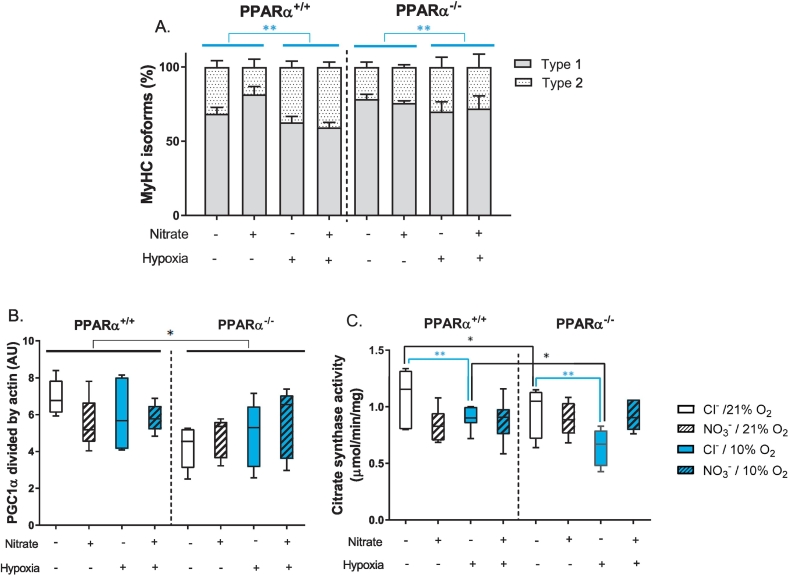
Soleus muscle morphology and mitochondrial markers. A) Myosin heavy chain isoform type proportions, B) PGC1α protein levels, C) Citrate synthase activity in mouse soleus. Data presented as mean ± SEM in graph A and as minimum to maximum values in graphs B and C, n = 5–10 per experiment group. *P < 0.05, **P < 0.01 genotype effect (black) or hypoxia effect (blue).

We sought to understand the effects of PPARα, nitrate-supplementation and hypoxic exposure, and any interactions between these factors, on relative expression of myosin heavy chain (MyHC) isoforms and mitochondrial biogenesis, dynamics and content. Relative expression of MyHC isoforms, suggested that hypoxic exposure induced a fibre-type shift in soleus towards a faster phenotype, with a 34% greater type II isoform expression compared with normoxic mice (*P* < 0.01, hypoxia main effect, Fig. 3A). Protein levels of peroxisome proliferator-activated receptor γ co-activator 1α (PGC1α), a master regulator of mitochondrial biogenesis, were 20% lower in PPARα^−/−^ mice compared with wild-types (*P* < 0.05, PPARα main effect, Fig. 3B), highlighting an effect of PPARα on PGC1α expression with no significant changes due to nitrate or hypoxia. Protein levels of mitofusin 2 (MFN2), a mediator of fusion between mitochondrial outer membranes, were the same in all mice (Supplementary Fig. 2).

Citrate synthase activity is a candidate marker of muscle mitochondrial density [45]. Activity of citrate synthase was 24% lower in the hypoxic chloride-treated mice compared with normoxic chloride-treated mice (*P* ≤ 0.01), however there was no difference in soleus citrate synthase activity between normoxic and hypoxic mice when treated with nitrate (Fig. 3C). Moreover, citrate synthase activity was 18% lower in the soleus of chloride-treated PPARα^−/−^ mice compared with that of chloride-treated wild-type mice, however no difference was seen between the PPAR^−/−^ and wild-type mice treated with nitrate. Thus, both hypoxia and PPARα knockout had a negative impact on soleus citrate synthase activity, but this was prevented by nitrate supplementation.

### 3.3 Mitochondrial respiratory function

Mitochondrial respiratory capacity was assessed in saponin-permeabilised soleus muscle fibres using two assays that employed substrate/inhibitor combinations to probe respiration under different coupling states and via different substrate-led pathways.

**Fig. 4:**
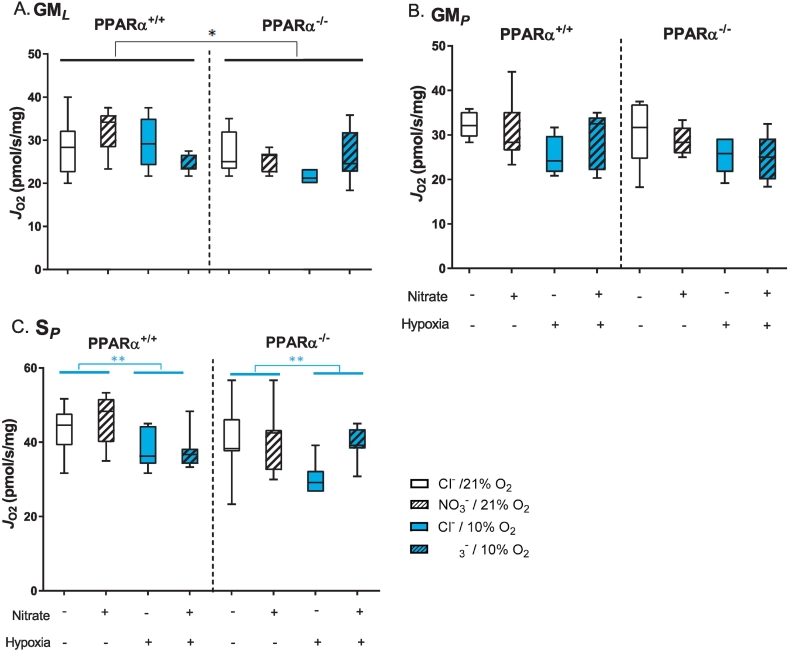
Mitochondrial respiratory capacities. A) LEAK state respiration (*J*_O2_) through the N-pathway via Complex I with malate and glutamate (GM_*L*_). B) OXPHOS state respiration (*J*_O2_) through the N-pathway via Complex I with malate, glutamate and ADP (GM_*P*_). C) OXPHOS state respiration (*J*_O2_) through the S-pathway via Complex II with succinate and rotenone (S_*P*_). Respiration rates are corrected to mass of soleus muscle fibres. Data are represented as minimum to maximum values, n = 6–10 per experiment group. *P < 0.05, **P < 0.01 genotype effect (black) or hypoxia effect (blue).

In the first assay, substrates for the N-pathway via Complex I and S-pathway via Complex II, were used to probe the effects of PPARα, nitrate-supplementation and hypoxia on the electron transfer system (ETS). LEAK state respiration in the presence of glutamate and malate (N-pathway via Complex I) was 12% lower in PPARα^−/−^ compared with wild-type mice (*P* ≤ 0.05, PPARα main effect, Fig. 4A), whilst neither nitrate nor hypoxia affected this. OXPHOS state respiration with glutamate and malate, was similar across all experimental groups (Fig. 4B). OXPHOS capacity in the presence of succinate and rotenone (S-pathway via Complex II) was 14% lower in hypoxic mice compared with normoxic mice (*P* < 0.01, hypoxia main effect, Fig. 4C). Thus, LEAK state respiration was suppressed by PPARα knockout, whilst OXPHOS respiration via the S-pathway was suppressed by hypoxia possibly due to a hypoxia-induced attenuation of Complex II activity.

**Fig. 5:**
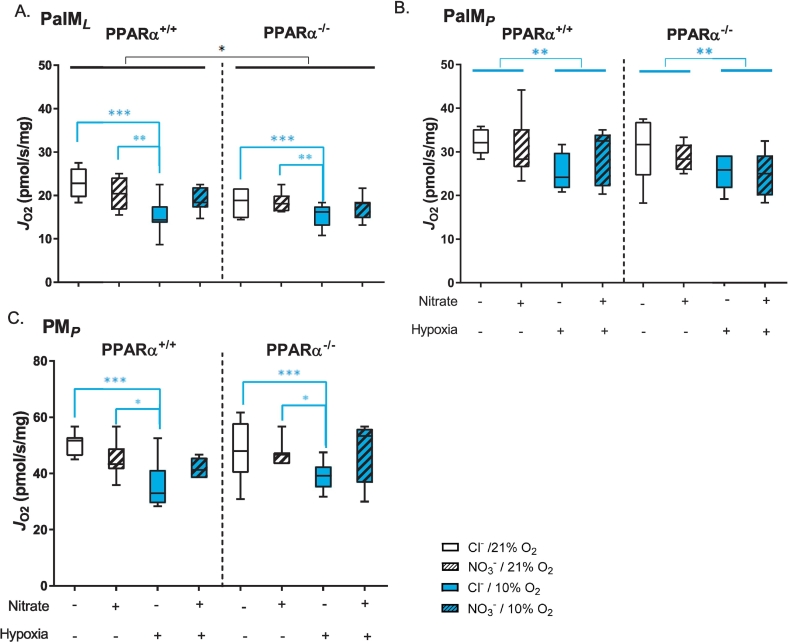
Mitochondrial fatty acid and pyruvate oxidation capacities. A) LEAK state respiration (*J*_O2_) with malate and palmitoyl carnitine (PalM_*L*_). B) OXPHOS state respiration (*J*_O2_) with malate and palmitoyl carnitine (PalM_*P*_). C) OXPHOS state respiration (*J*_O2_) through the N-pathway via Complex I with malate and pyruvate (PM_*P*_). Respiration rates are corrected to mass of soleus muscle fibres. Data are represented as minimum to maximum values, n = 6–10 per experiment group. *P < 0.05, **P < 0.01, ***P < 0.001 genotype effect (black) or hypoxia effect (blue).

In the second assay of mitochondrial respiratory function, fatty acid oxidation capacity was assessed in LEAK and OXPHOS states using palmitoyl-carnitine and malate as substrates, before pyruvate was added as a substrate for the N-pathway via Complex I. PPARα^−/−^ resulted in 10% lower LEAK state respiration (*P* < 0.05, PPARα main effect, Fig. 5A), though OXPHOS respiration was not affected (Fig. 5B). LEAK state respiration with palmitoyl-carnitine and malate was 26% lower in chloride-treated hypoxic mice compared with normoxic mice, but nitrate-supplementation prevented this (*P* < 0.001, Tukey's test of nitrate/hypoxia interaction, Fig. 5A). OXPHOS respiration supported by palmitoyl-carnitine was 15% lower in hypoxic mice compared with normoxic mice (*P* < 0.01, hypoxia main effect) and this was unaffected by nitrate treatment (Fig. 5B). OXPHOS respiration supported by pyruvate was 24% lower in chloride-treated hypoxic mice compared with normoxic mice (*P* < 0.001), however this was not seen in hypoxic mice supplemented with nitrate (Tukey's test of nitrate/hypoxia interaction, Fig. 5C). Taken together, these data suggest that (i) hypoxia lowers mitochondrial oxidation capacity for fatty acids and pyruvate, (ii) dietary nitrate prevents the hypoxic-induced decline in pyruvate (though not fatty-acid) oxidation, and (iii) these effects occur in a PPARα-independent manner.

**Fig. 6:**
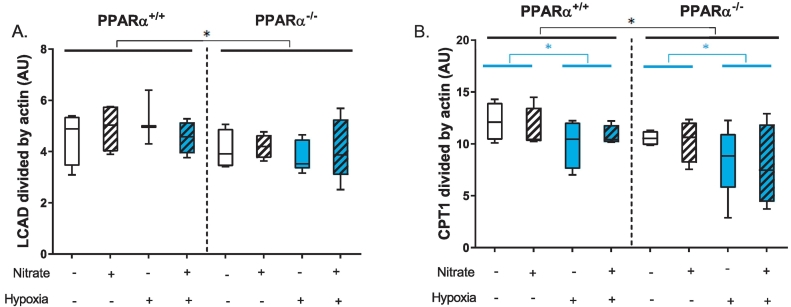
Fatty acid oxidation enzyme protein levels. A) Levels of long chain acyl CoA dehydrogenase (LCAD) and B) carnitine palmitoyltransferase protein in soleus muscle. Data are represented as minimum to maximum values, n = 3–5 per experiment group. *P < 0.05 genotype effect (black) or hypoxia effect (blue).

### 3.4 Expression of fatty acid oxidation enzymes

Finally, to further understand the PPARα-dependent effects of hypoxic exposure and nitrate supplementation on fatty acid and pyruvate oxidation we looked at expression of some key metabolic mediators. Levels of long chain acyl CoA dehydrogenase (LCAD) were 16% lower in PPARα^−/−^ mice compared with wild-type mice (*P <* 0.05, PPARα main effects, Fig. 6A). Similarly, protein levels of carnitine palmitoyltransferase 1 (CPT1) were 17% lower in PPARα^−/−^ mice compared with wild-type mice (*P <* 0.05, PPARα main effects, Fig. 6B). In addition, across both genotypes CPT1 levels were 17% lower in hypoxic mice compared with normoxic mice (*P* ≤ 0.05). These changes in levels of proteins regulated by PPARα were unaffected by nitrate treatment. Despite the effect of hypoxia on pyruvate-supported OXPHOS, and the counteracting effect of nitrate-supplementation, there were no differences in the levels of the regulatory protein pyruvate dehydrogenase kinase 4 (PDK4) between the groups (Supplementary Fig. 3).

## 4 Discussion

In the context of chronic tissue hypoxia, oxidative metabolism is suppressed [3], thereby matching tissue oxygen demand to the diminished supply. A key player in this response in a number of tissues is PPARα [5,7,8,10], a master regulator of fatty acid oxidation [4]. Dietary supplementation with inorganic nitrate exerts haemodynamic effects [29] which can improve tissue oxygenation, and alleviated the metabolic consequences of hypoxia in the rat heart [46]. Nitrate also stimulates oxidative metabolism in skeletal muscle, acting via PPARα [14], therefore we sought to investigate whether the protective effects of nitrate in the context of hypoxia were independent of PPARα.

In mouse soleus, hypoxia was associated with a fibre-type switch towards fast contracting fibres, with no effect of nitrate or PPARα expression. Citrate synthase activity was lower in the soleus with hypoxia and also with PPARα^−/−^, but in both cases this was prevented by dietary nitrate. Regarding mitochondrial respiration, LEAK state respiration with glutamate and malate and OXPHOS respiration with succinate were suppressed in hypoxic soleus. Meanwhile, fatty acid and pyruvate oxidation capacities were lower in hypoxic soleus, whilst nitrate reversed the hypoxia-induced decline in pyruvate (though not fatty-acid) oxidation. These effects on fatty acid and pyruvate oxidation occurred in a PPARα-independent manner. As expected, protein levels of LCAD and CPT1 were lower in PPARα^−/−^ mice compared with wild-types, but CPT1 levels were also lower in hypoxic mice across both genotypes, with no effect of nitrate treatment.

The complex study design allowed us to probe the interaction between the three factors, whilst addressing the core question of whether PPARα is necessary for the protective effects of nitrate on metabolism in hypoxic muscle. Central to this study was the statistical approach, which permitted us to examine interactions between nitrate, hypoxia and PPARα expression, and address the main hypothesis [44]. The three-way ANOVA represented a relatively conservative approach, and may have resulted in type II errors, particularly when considering the effect of a single stressor on an outcome, e.g. the effect of PPARα knockout on mitochondrial fatty acid oxidation capacity. Prior to commencing the study, we considered an alternative approach of separately examining wild-type and PPARα^−/−^ mice using two two-way ANOVAs, but rejected this approach because it fails to test the main hypothesis.

As with our previous studies [14,46], the precise control of nitrate intake was achieved with the use of a standardised quality-controlled diet and deionised water. A possible confounding factor was the negative effect of hypoxia on food and water intake, and thus body weight. This was most pronounced during the first week of hypoxic exposure, with water intake returning to normal in the second week and food intake recovering to match that of normoxic mice by the final week. The relatively long duration of the study was therefore a strength, since stabilisation of water intake meant that nitrate intake was identical between the supplemented groups for the final three weeks. A possible limitation of this study is the exclusive use of male mice, indeed dimorphic differences in the response to nitrate supplementation have been noted in human subjects [47]. Finally, our previous studies on nitrate supplementation in hypoxia have largely used rats, owing to the similarity in NO production rates [48] and circulating nitrate/nitrite levels between rats and humans [49], however it was necessary to use genetically-manipulated mice in this study in order to understand the role of PPARα.

In agreement with previous work on human muscle from our lab [6], hypoxia elicited metabolic responses in mouse soleus consistent with a decreased capacity for oxidative metabolism. The relative switch in abundance of myosin heavy chain isoforms away from type I MyHC towards type II MyHC, is consistent with a preference for glycolytic energy metabolism over oxidative phosphorylation, and this was paralleled by a lower activity of citrate synthase in hypoxic mouse soleus. Citrate synthase is a purported marker of mitochondrial content in human skeletal muscle [45], and fell in the muscle of lowlanders during an ascent of Everest alongside a loss of mitochondrial volume density [50]. Citrate synthase activity was also lower in the soleus of PPARα^−/−^ mice compared with wild-type mice. There was no difference in levels of PGC1α between hypoxic and normoxic mice though, suggesting that any changes in mitochondrial abundance in this study were not as a result of decreased PGC1α expression.

Further effects of hypoxia on oxidative metabolism were seen upon examination of mitochondrial respiratory capacity, including decreased OXPHOS respiration per unit mass of tissue in the presence of succinate (S-pathway via complex II), palmitoyl-carnitine and pyruvate, and lower LEAK state respiration with palmitoyl-carnitine. In previous studies from our group on rat soleus, we did not see any effect of hypoxia on mass-corrected respiration rates in permeabilised fibres despite some changes in metabolic gene expression, enzyme activities and muscle metabolite concentrations [10,14]. This may highlight a difference in the effect of hypoxia on rats and mice, but may have been due to the shorter duration of hypoxic exposure used previously (2 weeks). The lower respiratory rates we report here in hypoxic mouse soleus may have resulted from a loss of mitochondrial content per se, as suggested by lower citrate synthase activities. Succinate-supported OXPHOS capacity also fell in the muscle of lowlanders acclimatising to high-altitude though [6], whilst protein levels of succinate dehydrogenase were seen in a separate group of subjects undertaking the same ascent to Everest Base Camp [51]. Meanwhile, octanoyl-carnitine supported respiration rates declined in the muscle of lowlanders at high-altitude [6]. Moreover, in the present study protein levels of CPT1 were found to be lower in hypoxic mouse muscle than in normoxic mice, perhaps contributing to a lower fatty acid oxidation capacity, whilst *CPT1B* expression fell in human muscle at altitude [6]. Expression of *CPT1B* is controlled by PPARα, and our results may indicate a hypoxia-driven suppression of PPARα transcriptional activity, although notably in the present study hypoxic exposure resulted in lower CPT1 levels in both wild-type and transgenic mice, in addition to the negative effect of PPARα knockout.

In line with our hypothesis, dietary supplementation with inorganic nitrate elicited protective effects on metabolism in hypoxic mouse soleus independently of PPARα. The hypoxia-driven effects of lower citrate synthase activity and lower mitochondrial respiration rates (LEAK rate with palmitoyl-carnitine and OXPHOS rate with pyruvate) were prevented in nitrate-supplemented mice irrespective of genotype. However, it should be noted that not all effects of hypoxia were prevented by nitrate supplementation in this study, perhaps owing to the severity of the hypoxic exposure used here in comparison with previous studies [14,46]. Mechanistically, nitrate is known to exert effects on tissue oxygen delivery through its role as a vasodilator [27,28], and improvements in tissue oxygenation via enhanced blood flow may underpin the PPARα-independent effects reported here. In addition, nitrate exerts effects on skeletal muscle metabolism via other transcription factors, including PPARβ/δ [14]. Indeed, it has been suggested that high levels of expression of PPARβ/δ compensate for the loss of PPARα in the skeletal muscle of PPARα^−/−^ mice [52], and this may explain why mitochondrial fatty acid oxidative phosphorylation capacities were not lower in PPARα^−/−^ mice compared with wild-type mice in this study. Moreover, the activation of PPARα expression by nitrate is brought about by the sequential reduction of nitrate to nitrite and NO and thence the production of cGMP at the tissue level. cGMP also acts a mediator in the downstream cellular response to natriuretic peptides, eliciting numerous metabolic effects, many of which are independent of PPARα [53]. For instance, cGMP is known to promote mitochondrial biogenesis in skeletal muscle, acting via PGC1α [54]. Since PGC1α levels were unaffected in mouse muscle by hypoxia or by ablation of PPARα, it is possible that prevention of the hypoxia driven loss of citrate synthase activity seen here was a result of PGC1α activation by cGMP, brought about via improved NO availability.

## 5 Conclusions

In conclusion, dietary nitrate is able to exert effects on skeletal muscle metabolism that counteract the effect of hypoxia, and this occurs independently of PPARα. Our work may hold implications for the use of nitrate in the treatment of diseases in which hypoxia is a factor and in which PPARα expression and/or transcriptional activity is attenuated. Moreover, our work suggests nitrate supplementation might offer benefits to lowlander subjects at high-altitude, in whom PPARα transcriptional activity is suppressed [6], and suggests that the high circulating nitrogen oxide levels seen in some Tibetan populations [32,34] might offer metabolic benefits despite lower PPARα expression in the muscles of some groups, e.g. the Sherpa [6].

The following are the supplementary data related to this article.

Supplementary Fig. 1 Daily food intake (n = 10–12 per group in pair-housed cages) averaged over each week, with nitrate supplementation beginning on week 1 and hypoxic treatment beginning on week 2. Data represented as mean ± SEM. Supplementary Fig. 1

Supplementary Fig. 2 Levels of mitofusin 2 (MFN2) protein in soleus muscle. Data are represented as minimum to maximum values, n = 4–5 per experiment group. Supplementary Fig. 2

Supplementary Fig. 3 Levels of pyruvate dehydrogenase kinase 4 (PDK4) protein in soleus muscle. Data are represented as minimum to maximum values, n = 4–5 per experiment group. Supplementary Fig. 3

## Availability of supporting data

The data sets supporting the results of this article are available in the University of Cambridge repository. Details to follow upon acceptance of manuscript.

## Abbreviations

cGMP: cyclic guanosine monophosphate
CPT: carnitine palmitoyltransferase
HIF: hypoxia-inducible factor
LCAD: long chain acyl CoA dehydrogenase
LEAK: leak state respiration, in the absence of ADP
MFN2: mitofusin 2
OXPHOS: oxidative phosphorylation
PGC1α: peroxisome proliferator-activated receptor gamma co-activator 1α
PPARα: peroxisome proliferator-activated receptor alpha
PDK: pyruvate dehydrogenase kinase

## Competing interests

The authors declare that they have no competing interests.

## Transparency document

Transparency document Image 1
