# Supplementary figures and images for: PPARα-independent effects of nitrate supplementation on skeletal muscle metabolism in hypoxia
Source: Biochim Biophys Acta Mol Basis Dis. 2019 Apr 1;1865(4):844–53. doi: 10.1016/j.bbadis.2018.07.027 (PMC6414754; doi:10.1016/j.bbadis.2018.07.027)

Supplementary Figure 1

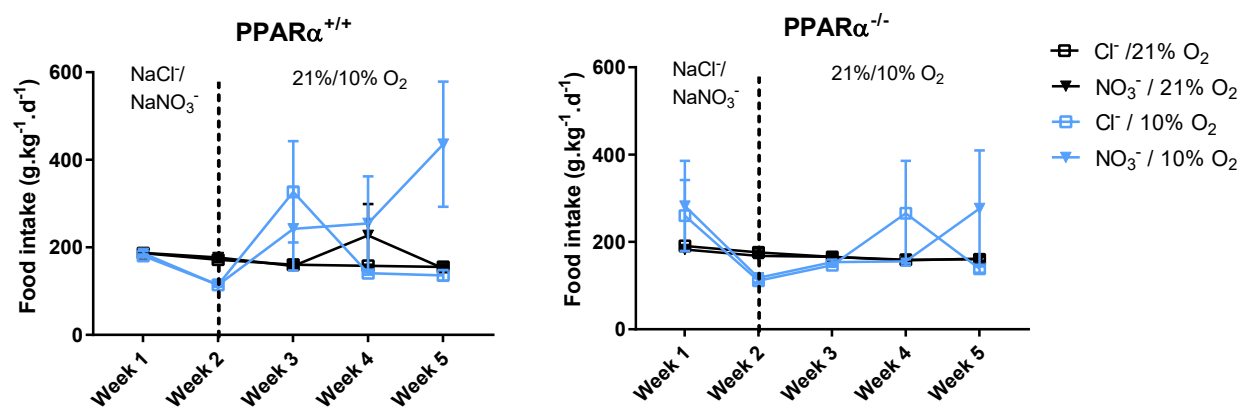

Supplement: Supplementary Fig. 1 — Daily food intake (n = 10–12 per group in pair-housed cages) averaged over each week, with nitrate supplementation beginning on week 1 and hypoxic treatment beginning on week 2. Data represented as mean ± SEM. [file mmc1.pdf]

Supplementary Figure 2

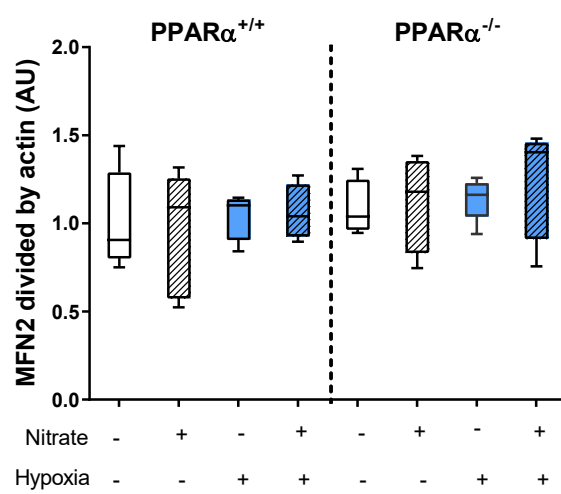

Supplement: Supplementary Fig. 2 — Levels of mitofusin 2 (MFN2) protein in soleus muscle. Data are represented as minimum to maximum values, n = 4–5 per experiment group. [file mmc2.pdf]

Supplementary Figure 3

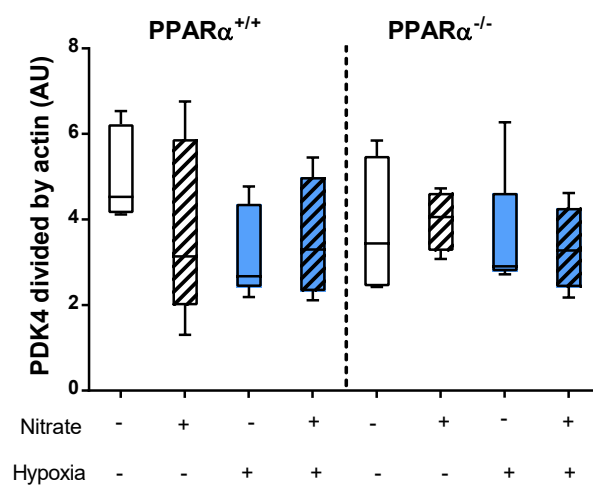

Supplement: Supplementary Fig. 3 — Levels of pyruvate dehydrogenase kinase 4 (PDK4) protein in soleus muscle. Data are represented as minimum to maximum values, n = 4–5 per experiment group. [file mmc3.pdf]
